# Copper nanoparticles biosynthesis by *Priestia megaterium* and its application as antibacterial and antitumor agents

**DOI:** 10.1038/s41598-024-72598-3

**Published:** 2024-10-09

**Authors:** Salma H. Mohamed, Badawi A. Othman, Basma T. Abd-Elhalim, Mohammed N. Abou Seada

**Affiliations:** Department of Agricultural Microbiology, Faculty of Agriculture, Ain Shams University, Hadayek Shoubra, PO Box 68, Cairo, 11241 Egypt

**Keywords:** Agro-industrial wastes, Antibacterial effect, Antitumor activity, Copper nanoparticles, Cytotoxicity activity, Caco-2-HTB-37 cell line, *Priestia megaterium*, Biomaterials, Industrial microbiology, Nanobiotechnology, Biotechnology, Nanoscience and technology

## Abstract

The growth of material science and technology places high importance on creating better processes for synthesizing copper nanoparticles. Thus, an easy, ecological, and benign process for producing copper nanoparticles (CuNPs) has been developed using *Priestia* sp. bacteria utilizing a variety of low-cost agro-industrial wastes and byproducts. The biosynthesis of CuNPs was conducted using glucose medium and copper ions salt solution, then it was replaced by utilizing low-cost agro-industrial wastes. UV–visible spectroscopy, dynamic light scattering (DLS), X-ray diffraction (XRD), High-resolution transmission electron microscope (HR-TEM), Attenuated Total Reflectance and Fourier transform infrared (ATR-FTIR), and zeta potential were used to characterize the biosynthesized CuNPs. The cytotoxicity of CuNPs using Vero -CCL-81 cell lines, and antibacterial and antitumor properties using human colon epithelial colorectal adenocarcinoma Caco-2-HTB-37 cell lines were assessed. The UV–visible and DLS studies revealed CuNPs formation, with a maximum concentration of 6.19 ppm after 48 h, as indicated by a 0.58 Surface plasmon resonance (SPR) within 450 nm and 57.73 nm particle size. The 16S rRNA gene analysis revealed that *Priestia* sp. isolate is closely related to *Priestia megaterium* and has been deposited in the NCBI GenBank with accession number AMD 2024. The biosynthesis with various agro-industrial wastes indicated blackstrap sugar cane molasses being the most effective for reducing CuNPs size to 3.12 nm owing to various reducing and stabilizing active compounds. The CuNPs were free of contaminants, with a sphere-shaped structure and a cytotoxicity assessment with an IC_50_ of 367.27 μg/mL. The antibacterial activity exhibited by the most susceptible bacteria were *Bacillus cereus* ATCC 11788 and *Staphylococcus aureus* ATCC 6538 with inhibition zones of 26.0 mm and 28.0 mm, respectively. The antitumor effect showed an IC_50_ dose of 175.36 μg/mL. Based on the findings, the current work sought to lower product costs and provide a practical solution to the environmental contamination issues brought on by the buildup of agricultural wastes. In addition, the obtained CuNPs could be applied in many fields such as pharmaceuticals, water purification, and agricultural applications as future aspects.

## Introduction

Since the size of nanoparticles (NPs) sits between that of their bulk materials, which are usually larger than 100 nm, and their nanoscale phenomena, many researchers are interested in studying the synthesis of NPs. The type, shape, and size of nanoparticles (NPs) often influence their characteristics, according to prior research by^1,2^.

Because of their unique physiological characteristics that set them apart from their bulk materials—such as their active surface area, chemical reactivity, hardness, diffusivity, biological activity, electrical conductivity, resistivity, and miniaturized size distribution (measured in nanometers)—metallic nanoparticles in particular have attracted a lot of attention^2^.

The creation of nanoparticles by physical and chemical methods is still expensive and necessitates the use of dangerous substances. Consequently, there is increased concern about creating straightforward, affordable, and useful solutions. Green synthesis, according to^3^, refers to innovative, economical, environmentally benign, and less hazardous procedures. According to^4^ green synthesis is an alternative to chemical synthesis that uses plant extracts or microorganisms. According to their composition, size, shape, and other factors, nanomaterials come in a variety of forms^5,6^. There is little variation in the biosynthesis of metal nanoparticles derived from different sources. For example, several methods produce metal nanoparticles with distinct properties, as noted by^4^.

As an eco-friendly, inexpensive, and straightforward substitute for dangerous, costly, and time-consuming chemicals and physical methods, a great deal of research is focused on the biosynthesis of copper nanoparticles (CuNPs) with antimicrobial properties, which is mediated by various natural sources such as extracts of plant parts (root, stem, leaves, seeds), algae, bacteria, and fungi^1–5^.

It was stated by^7^ that bacteria are chosen to synthesis NPs because of a number of benefits, including quick production, ease of culture, high stability, resistance to the majority of dangerous heavy metals, and the potential to produce sustainable NPs on a large scale. According to^8^, microorganisms are recommended to synthesis NPs because of their great capacity to remove heavy metal ions. Furthermore, metabolites that can reduce metals are gathered during the stationary phase of their growth, when metabolic stress reaches its highest, and biosynthesis occurs when the presence of metal ions in the growth environment results in the generation of NPs. Therefore, it seems sense to assume that by creating NPs by combining those molecules with proteins, polysaccharides, and other metabolites, microorganisms can reduce the toxicity of metals^7^.

The literature explores biomolecules and polysaccharides as capping, chelating, and reducing agents in nanoparticle synthesis, aiming to prevent aggregation, extend stability, and enhance antibacterial and antifungal activity^1,3,4^.

According to^9^, Zeta potential, high-resolution transmission electron microscopy with energy dispersive X-ray (HR-SEM–EDX), dynamic light scattering (DLS), Fourier transform infrared (FTIR), UV–visible spectroscopy, and other techniques are commonly used to characterize the physiochemical properties of nanoparticles.

The fabrication of copper nanoparticles is preferred over other nanoparticles because copper is plentiful, less expensive than other metals like gold, silver, and platinum, and less poisonous than other nanomaterials like silver nanoparticles (AgNPs)^1,4^. Owing to these characteristics, CuNPs have shown up in a number of fascinating applications across a wide range of industries, including electronics, environmental protection, cosmetics, pharmaceuticals, and biomedicine. As many reports discussed, many antimicrobial, antiproliferative, antioxidant, and anticancer properties exist for many varieties of nanoparticles^1–10^.

A variety of agro-industrial wastes are used in the work by^10^ for NPs biosynthesis in low-cost medium because they are cheap, abundant, and sustainable sources of carbon or nitrogen.

Through the use of *Priestia megaterium* bacteria, this work aimed to biosynthesize CuNPs from various agro-industrial wastes and byproducts in order to lower product prices and offer a workable solution to the problems with environmental contamination caused by the accumulation of agricultural wastes.

## Materials and methods

### Strains and isolate collection and standard inocula preparation

The isolate of *Priestia* sp. was obtained from soil cultivated with a broad bean (*Vicia faba*) and was deposited in the culture stook at the Agricultural Microbiology Department, Faculty of Agriculture, Ain Shams University, Cairo, Egypt. Also, it provided four pathogenic strains of *Staphylococcus aureus* ATCC 6538, *Bacillus cereus* ATCC 11788, *Escherichia coli* ATCC 8379, and *Pseudomonas aeruginosa* ATCC 27853. The isolate was kept at 4 °C until it was needed again by occasionally subculturing it on nutrient agar medium. *Priestia* sp. standard inoculum was made in accordance with^11^. An individual loop of the strain under examination was introduced into a 250 mL conical flask that held 50 mL of nutritional broth medium^12^. Next, let it incubate at 30 °C for 24 h. The standard inoculum was one milliliter (mL) of the culture that contained 7.03 × 10^7^ CFU/mL. A standard inoculum was generated for pathogenic bacterial strains in compliance with^13^ guidelines. 50 mL of glucose broth were used to incubate a loop of the active bacterial culture for 24 h at 37 °C in shake flasks spinning at 150 rpm.

### Copper sulfate solution preparation

Copper sulfate (CuSO_4_) solution with a concentration of 1.0 mM was made by weighing 0.25 g of CuSO_4_, adding it to 1000 mL of sterile double-distilled water, and then passing the mixture through an aseptic 0.22 mm filter^14^.

### Identification of *Priestia* sp. isolate

*Priestia* sp. isolate was confirmed by the 16S rRNA sequencing in reference to^15^. The partial 16S rRNA gene sequence was aligned, analyzed in comparison with several nucleotide information, using the BLAST application provided in the NCBI GenBank. For discovering the closest homologous bacterial members, data was retrieved through constructing a phylogenetic tree using the Neighbor-joining method.

### Agro-industrial wastes collection

For the CuNPs biosynthesis process, six different local agro-industrial wastes and by-products were utilized. As shown in (Table S1), these wastes and byproducts were gathered from various sources.

### Pretreatment of agro-industrial wastes and byproducts

After being cleaned of any contaminants using warm water and tap water, the used agro-industrial wastes, and by-products such as sugar beet waste, banana peels, and sugar cane bagasse were pulverized, and oven dried for a whole night at 50 °C. Subsequently, the dehydrated substrates were ground using a lab grinder, sieved to remove big particles and reach a size of 5.0 mm, and stored for next studies to create the powdered form^16^. In order to precipitate the undesirable metal salts, blackstrap sugar beet and sugarcane molasses were diluted 1:1 with water until the acidity reached pH 4.0, heated for one hour at 100 °C in a water bath, and then left overnight^17^. The pH of the whey from Arish cheese was adjusted to 4.5 using 5 N HCl, and the proteins were then heated to 121 °C for 15 min to denature them. This was followed by a 15-min centrifugation at 10,000 rpm^18^.

### Biosynthesis of CuNPs by *Priestia* sp. using standard fermentation medium (glucose broth)

CuNPs' biosynthesis has been proven, as reported by^19,20^. A 250 mL Erlynmner flask was filled with a one milliliter inoculum of *Priestia* sp. together with 50 ml of glucose liquid (broth) medium^11^. CuNPs biosynthesis was induced in the infected flasks by shaking them at 150 rpm for 30 °C in a shaking incubator (Shin Saeng, South Korea). 50 ml of sterilized 1 mM CuSO_4_.5H_2_O solution was added aseptically after the initial 12-h incubation period, and the mixture was then incubated for a further 48 h under identical conditions. The broth medium's hue shift signaled the detection of CuNPs production. Following the completion of the CuNPs manufacturing process, the broth culture was centrifuged (using a SIGMA 2–16 P centrifuge) for 10 min at 10,000 rpm. The biosynthesized CuNPs were assessed by collecting the cell-free filtrate. The collected supernatant was dried at 60 °C for 24 h to obtain the nanoparticles in a powdered form.

### Biosynthesis of CuNPs by *Priestia* sp. using agro-industrial wastes and byproducts

Arish cheese whey, banana peel, blackstrap sugar beet molasses, blackstrap sugar cane molasses, sugar beet waste, and sugarcane bagasse were added with a similar concentration of the major carbon source for the fermentation medium (glucose) in order to investigate and select the most suitable agro-industrial wastes and byproducts. The following compounds' total carbon contents were collected from the Central Laboratory of the Agriculture Research Center in Giza, Egypt: 5.0, 30.0, 60.0, 53.0, 62.5, and 51.0%, respectively. One milliliter inoculum of *Priestia* sp. was inoculated into a 50 ml of the agro-industrial prepared broth medium for each source and incubated at 150 rpm for 30 °C in a shaking incubator. The biosynthesized CuNPs were assessed by collecting the cell-free filtrate as mentioned previously.

### The time course of the biosynthesized *Priestia* sp. CuNPs (P-CuNPs)

To determine the ideal incubation period needed for P-CuNPs synthesis, the time history of the biosynthesized P-CuNPs was assessed. This involved collecting 5 ml of growing supernatant at various incubation times every 24 h, centrifuging it at 10,000 rpm for 10 min aseptically, and estimating the quantities of P-CuNPs collected supernatant using an AAS Shimadzu Atomic Absorption Spectrophotometer (AA-6300, USA) at Creative Egyptian Biotechnologists (CEB) company, Dokki, Giza, Egypt, complementary experiments.

### Characterization of the biosynthesized *Priestia* sp. CuNPs (P-CuNps)

A visible shift in hue indicated the initial identification of *Priestia* sp. CuNPs production. Using UV–vis spectroscopy (JASCO Corp., V-570, USA) at wavelengths between 400 and 700 nm on the nanoparticles collected supernatant, the main investigation for CuNPs formation was found. Using an X-ray diffractometer (Shimadzu -7000, UK) at the Egyptian Petroleum Research Institute (EPRI), Cairo, Egypt, powder XRD analysis was used to determine the crystalline states of powdered CuNPs. According to the Joint Committee on Powder Diffraction Standards JCPDS: (80-1917 and 78-2076), the crystalline nature of the produced Cu nanoparticles with XRD analysis was well matched with monoclinic phase of Cu. The XRD pattern can be indexed to metallic copper nanoparticles (CuNPs) and different mixed copper oxides (CuO, Cu_2_O, and Cu_4_O_3_) nanoparticles^20^. Dynamic light scattering was used to determine particle size and zeta potential for CuNPs suspension. The structural morphology of CuNPs in the collected solution was characterized at 80 kV at The Regional Center for Mycology and Biotechnology (RCMB), Al-Azhar University, Cairo, Egypt, using a JEOL JEM-1010 high resolution transmission electron microscope (JEOL, Tokyo, Japan). Attenuated Total Reflectance and Fourier transform infrared (ATR-FTIR) (THERMO NICLOT, 50, USA) to identify the presence of functional groups linked to the P-CuNPs solution^3^.

### Application of P-CuNPs as antibacterial agent

Four pathogenic bacterial strains—two Gram positive (G + ve) and two Gram negative (G-ve)—were used to assess the biosynthesized P-CuNPs antibacterial efficacy. On Mueller–Hinton agar medium^11^, the following bacteria were cultured: *S. aureus* ATCC 6538, *B. cereus* ATCC 11788, *E. coli* ATCC 8739, and *P. aeruginosa* ATCC 21823. The inhibitory impact of six different doses of *Priestia* sp. CuNPs against the four bacterial strains was tested using the well diffusion technique^21,22^. In short, 1 mL of each bacterial inoculum was added to petri dishes, and then medium was added. Using a 7 mm diameter sterilized cork borer, wells were created and then filled with 0.1 mL of P-CuNPs dispersed in distilled water at various doses (1000, 500, and 250, 125, 62.5, and 31.25 μg/mL). The kanamycin was the control antibiotic with concentration of 1000 µg/mL. For 24 h, each plate was incubated at 37 °C. Using a ruler, the zone of inhibition surrounding each well was measured and represented as IZD (mm)^23^. The activity index was determined by comparing the diameter of the inhibition zone of P-CuNPs with the standard reference antibiotic using the following equation, as per the methods described in^24–26^:

1 $$Activity\, index \,\left(AI\right)= Inhibition \,zone \,diameter \,\left(IZD\right)\,of \,PCuNPs/ Inhibition\,zone \,diameter\, \left(IZD\right) \,of \,reference \,antibiotic$$

### P-CuNPs cytotoxicity and application as antitumor agent

At Science Way Company in Cairo, Egypt, the cytotoxicity, and anticancer activity of P-CuNPs were evaluated against human colon epithelial colorectal adenocarcinoma Caco-2-HTB-37 cell lines and kidney epithelial normal ccl-81 cell lines, respectively. In accordance with the protocol outlined by^27^, a full monolayer sheet was developed by incubating the 96-well tissue culture plate at 37 °C for 24 h after inoculation with 1 × 10^5^ cells/ml (100 µl/well). Once a confluent sheet of cells had formed, the growth material was removed from the 96-well micro titer plates and the cell monolayer was twice washed with wash media. P-CuNPs were divided into twofold dilutions in RPMI medium containing 2% serum (maintenance medium), and 0.1 mL of each dilution was examined in several wells, leaving. Physical indicators of toxicity, such as shrinkage, rounding, granulation, or partial or whole loss of the monolayer, were examined in the cells. Twenty microliters of MTT (3-(4,5-dimethylthiazol-2-yl-)-2,5 diphenyltetrazolium bromide) solution were added to each well after the solution (5 mg/ml in phosphate buffer solution) was prepared (Bio basic Canada Inc.). Media were set on a shaking table and shake at 150 rpm for five minutes to fully incorporate the MTT. For four hours, incubate at 37 °C with 5% CO_2_ to allow the MTT to metabolize. If required, remove the media, and dry the plate with paper towels to get rid of any residue. Formazan, an MTT metabolic product, may be redissolved in 200 µL DMSO. To completely combine the formazan and solvent, it was set on a shaking table and shook for five minutes at 150rpm. Optical density was obtained at 560 nm and subtract background at 620 nm. Optical density should be directly correlated with cell quantity.

2 $$\% Cell \,viability=(Mean \,Abs \,control-Mean \,Abs \,P.CuNPs )/(Mean \,Abs \,control) \, \times\,100$$

where: Abs absorbance at 560 nm.

### Statistical analysis

Using IBM® SPSS® Statistics software, the findings were statistically examined and reported as means (2017). The A *P*-value of 0.05 was used for Duncan's test^28^. The biocompatibility and anticancer results calculated as IC_50_ were reported as mean ± SD and the difference between the groups was tested using two-way ANOVA, using Graph Pad Prism 8.4.1 (GraphPad Software, San Diego, CA, www.graphpad.com) and the interaction was found significant as *P* < 0.05. All experiments were carried out as n = 3.

## Results

### Biosynthesis of P-CuNPs using glucose broth medium

#### Color change observation

**Fig. 1:**
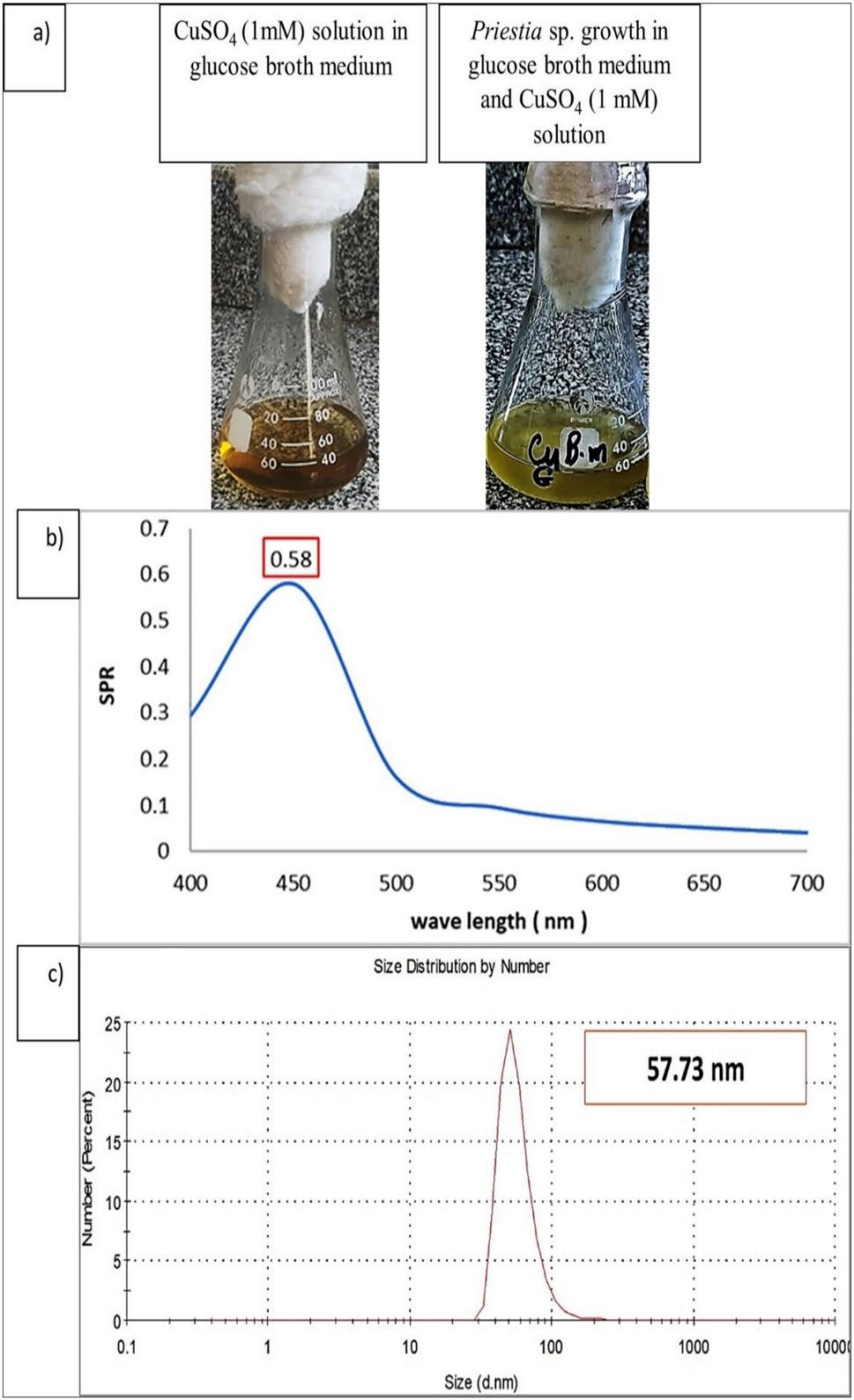
(**a**) The alteration in color, signifying the production of *Priestia* sp. CuNPs changed the mixture's reaction color from pale yellow to greenish, (**b**) The SPR spectrum at 0.58, and (**c**) DLS characterization for CuNPs formation after 48 h at 30 °C using shaking flasks at 150 rpm.

As seen in Fig. 1a. After 48 h of incubation at 30 °C, 150 rpm, and shaking, the reaction mixture color of *Priestia* sp. combined with 1mM CuSO_4_ solution (copper ions source) changed from light yellow to greenish.

#### UV–visible spectroscopy and DLS characterization

The findings obtained from the study of the culture supernatant at wavelengths between 400 and 700 nm using UV–vis spectroscopy to analyze P-CuNPs creation are displayed in (Fig. 1b). The highest SPR peak for the reaction mixture solution occurred at λ max 450 nm and was 0.58. The size of the biosynthesized P-CuNPs was 57.73 nm, according to data in (Fig. 1c).

### Identification of *Priestia* sp. isolate

**Fig. 2:**
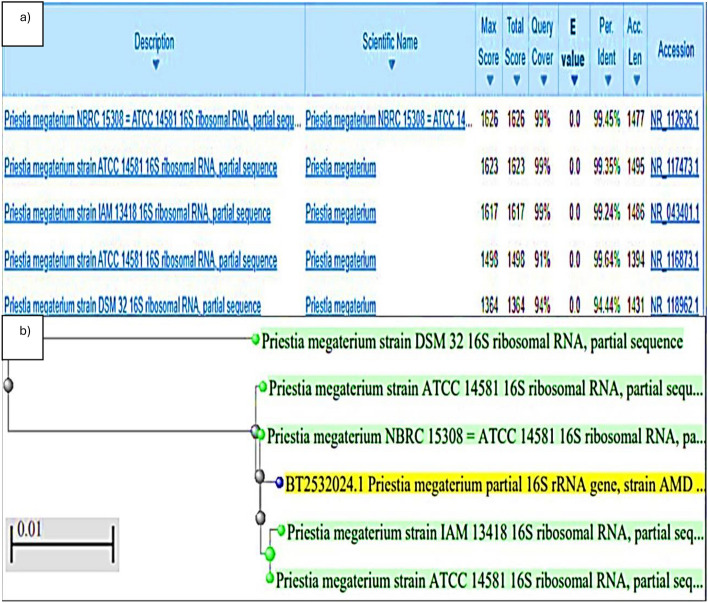
(**a**) Match score for *Priestia* sp. isolate showing 99% similarity to *Priestia megaterium*, (**b**) Phylogenetic-tree of *Priestia megaterium*.

Results of the 16s rRNA gene sequence analysis of *Priestia* sp. isolate shows that it belongs to Genus *Priestia* sp., closely related to *Priestia megaterium* (99% similarity) Fig. 2(a), after analysis and comparison with nucleotide sequences of NCBI as shown in Fig. 2(b).

### The efficiency of different agro-industrial wastes and byproducts on the biosynthesis of P-CuNPs

**Fig. 3:**
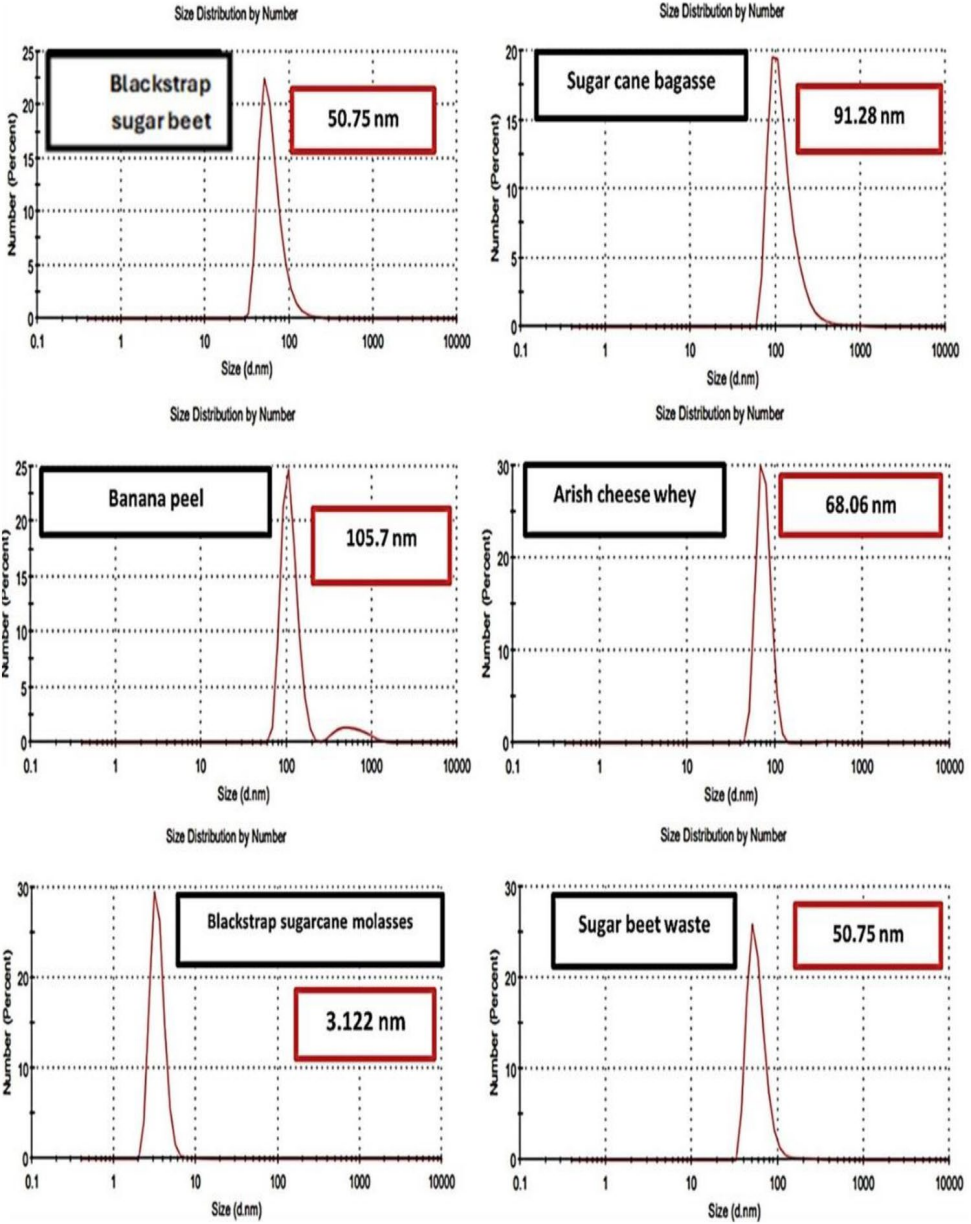
Dynamic light scattering (DLS) characterization of biosynthesized P-CuNPs diameter size by *P. megaterium*, affected by various agro-industrial wastes and byproducts after 48 h at 30 °C using shaking flasks at 150 rpm.

It was clear from (Fig. 3) that the agro-industrial wastes and byproducts were useful in the biosynthesis process when it came to the DLS characterisation for size investigation of the biosynthesized P-CuNPs. The P-CuNPs with arish cheese whey, banana peel, blackstrap sugar cane molasses, blackstrap sugar beet molasses, sugar beet waste, and sugarcane bagasse had relative sizes of 68.06, 105.7, 3.122, 50.75, 50.75, and 91.28 nm, respectively.

### The time course of P-CuNPs biosynthesis

**Fig. 4:**
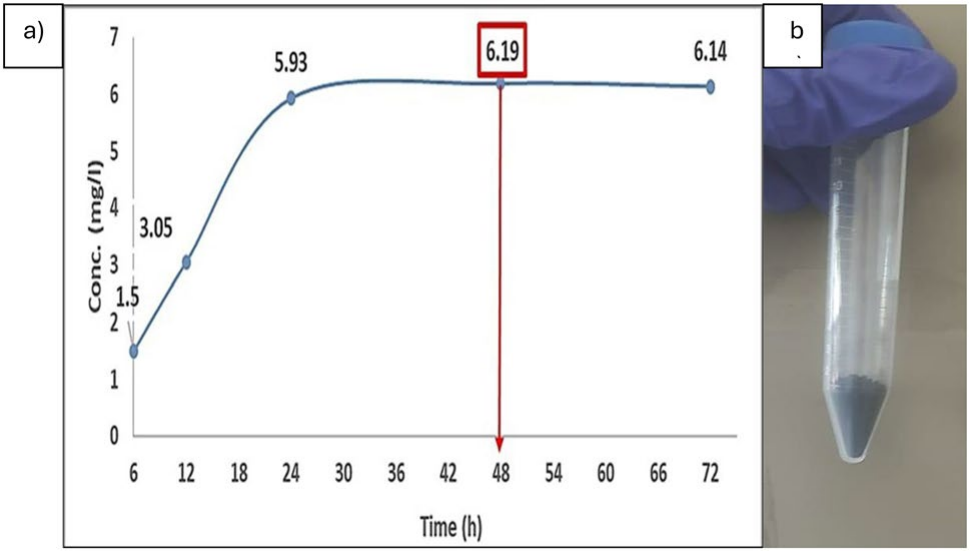
(**a**) Time course for P-CuNPs biosynthesized using blackstrap sugarcane molasses at 30 °C using shaking flasks at 150 rpm , (**b**) The obtained powder of P-CuNPs after optimization with blackstrap sugarcane molasses.

The biosynthesis time course for P-CuNPs was ascertained by computing the concentration of the biosynthesized CuNPs in the cell-free extract. 48 h was the ideal reaction time to obtain a high concentration of P-CuNPs, as (Fig. 4a) illustrates. As seen in Fig. 4b. the obtained P-CuNPs after optimization using blackstrap sugar cane molasses.

### Characterization of the biosynthesized P-CuNPs utilizing the best agro-industrial waste (blackstrap sugarcane molasses)

#### Transmission *electron* microscope (TEM) analysis

**Fig. 5:**
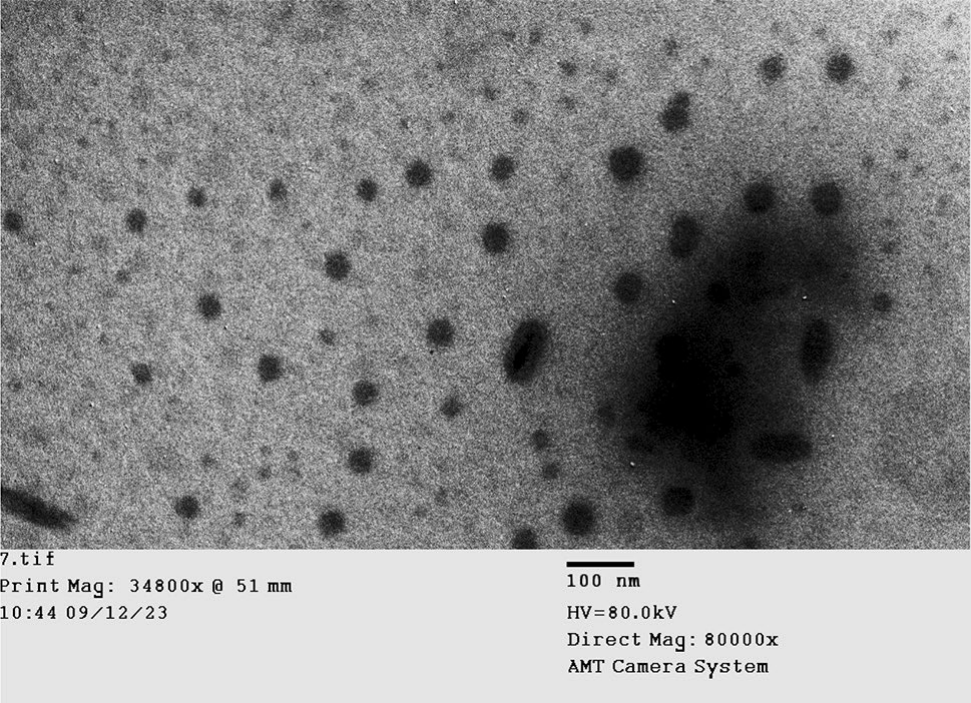
TEM characterization of P-CuNPs utilizing blackstrap sugarcane molasses after 48 h at 30 °C in shaking flasks at 150 rpm.

Utilizing a transmission electron microscope, the morphology of the CuNPs generated by *P. megaterium* was examined. Cu nanoparticles are sphere particles coated with protein and exhibit good desperate behavior, as seen in (Fig. 5).

#### X-ray diffractometer (XRD)

**Fig. 6:**
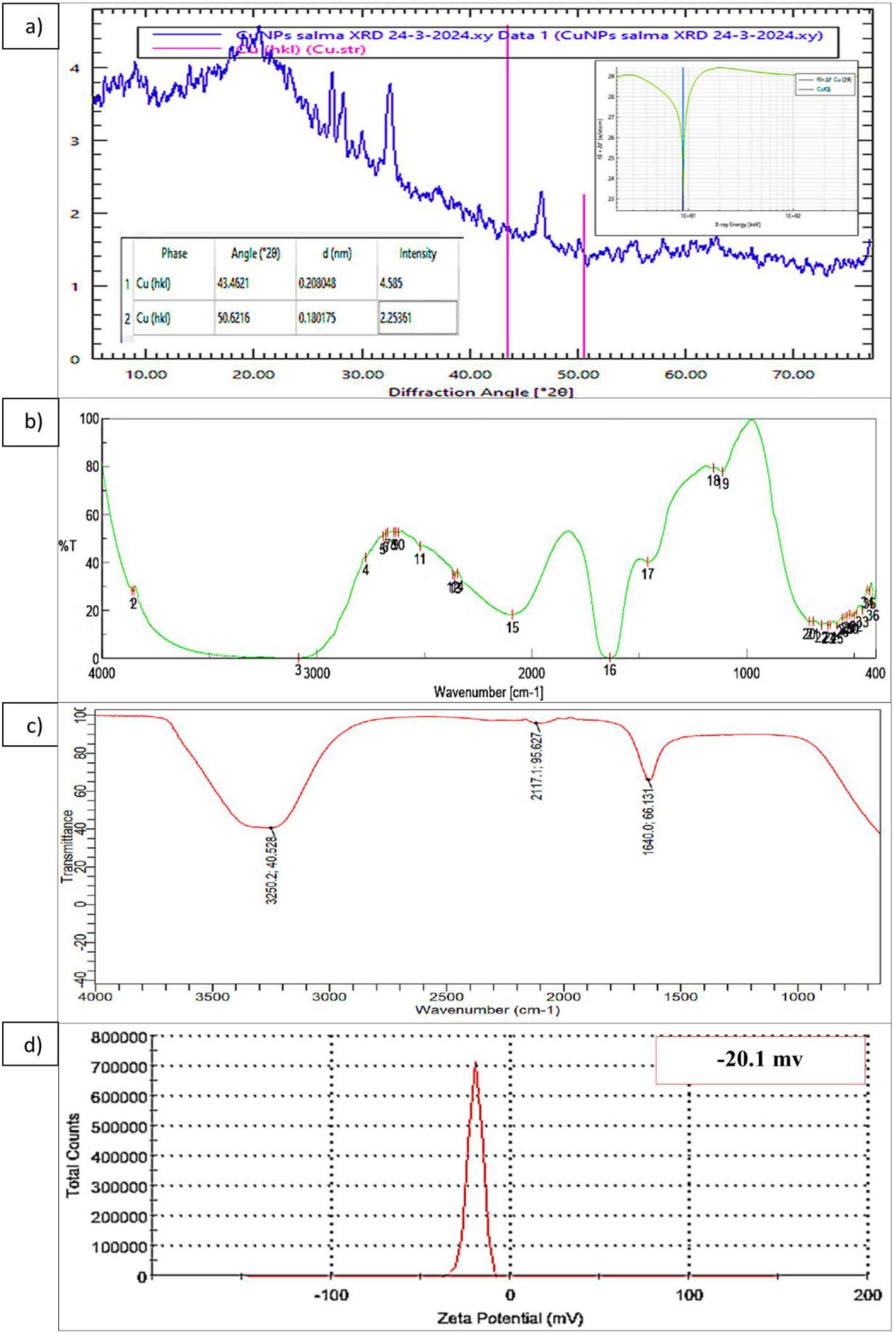
(**a**) X-ray diffractometer (XRD) characterization of P-CuNPs, (**b**) The FTIR analysis of the active compounds responsible for the reduction, coating, and stabilization of P-CuNPs in *P. megaterium* cell free extract, and (**c**) The FTIR analysis P-CuNPs, (**d**) Zeta potential analysis of P-CuNPs using blackstrap sugarcane molasses after 48 h at 30 °C using shaking flasks at 150 rpm.

The results of the XRD analysis revealed that the P-CuNPs' XRD pattern had two distinct peaks at 43.46° and 50.62°, with intensities of 4.58 and 2.25, respectively. No further peaks were found, as shown in (Fig. 6a). Crystalline nature of the produced P-CuNPs with XRD confirmed according to Joint Committee on Powder Diffraction Standards JCPDS: (80-1917 and 78-2076) was well matched with monoclinic phase of Cu.

#### Attenuated total reflectance Fourier transmission infrared (ATR-FTIR) characterization

The cell-free extract of *P. megaterium* showed a range of 400–4000 cm^-1^ functional group presence, as shown in (Fig. 6b). Peak locations were found to be between 412.692 and 3860.79 cm^-1^. According to the findings, the active functional groups are C-H, O–H, O = C = O stretching, N = C = S stretching, N–H bending, C-H bending, C–N stretching, C–CL, C–Br, and C-L stretching. These groups, in turn, correspond to the following compounds: halo compound, benzene derivative, alkene, alcohol, carboxylic acid, carbon dioxide, and isothiocyanate. As shown in Fig. 6c the FTIR analysis showed three distinct peaks harbored in 1640.02865, 2117.12789, and 3250.23859 which correspond to N–H bending, N = C = S stretching isothiocyanate, and O–H stretching.

#### Zeta potential analysis

As can be shown in Fig. 6d, the zeta potential for P-CuNPs was determined to be -20.1 mv.

### Application of P-CuNPs as antibacterial agent

**Fig. 7:**
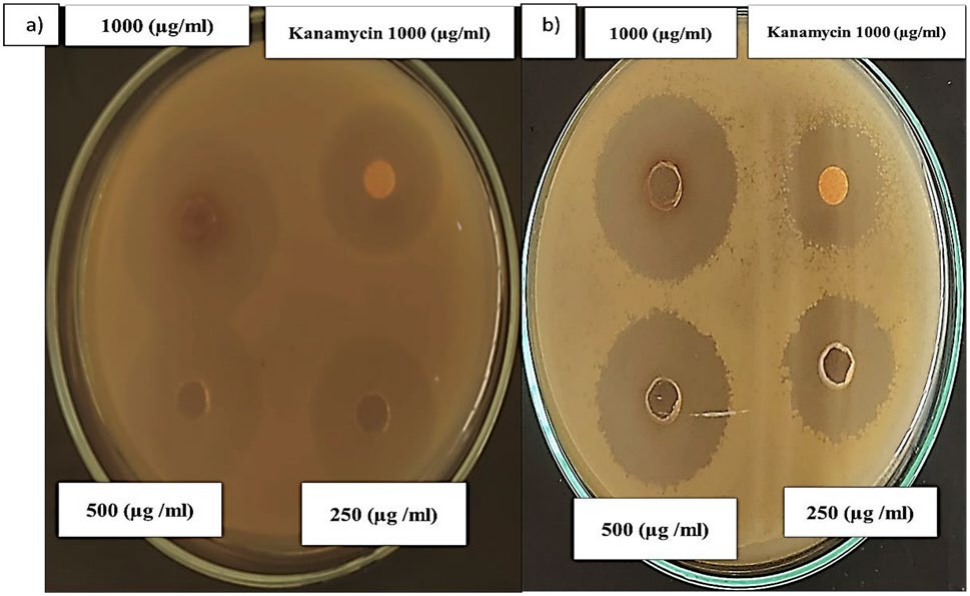
Antibacterial activity of P-CuNPs against the most sensitive pathogenic strains, (**a**) *S. aureus*, (**b**) *B. cereus*.

A test for the biosynthesized P-CuNPs' antibacterial susceptibility was conducted using four pathogenic bacteria, two of which were gram positive and the other two gram negative, along with an antibiotic as a control. The most vulnerable bacteria were *S. aureus* and *B. cereus* with 28.0 mm and 26.0 mm (IZD) and activity index (AI) values of 2.0 and 1.54, respectively, according to data in Table S2. In contrast, *P. aeruginosa* growth was unaffected compared to *E. coli*, which had the lowest susceptibility with a minimum IZD of 12 mm and AI of 0.86. Since (Fig. 7) eliminated the antibacterial activity of gram positive infections, it was shown that P-CuNPs had a significantly increased antibacterial impact against gram-positive pathogens as opposed to gram-negative pathogens.

### P-CuNPs cytotoxicity and application as antitumor agent using (MTT) assay

**Fig. 8:**
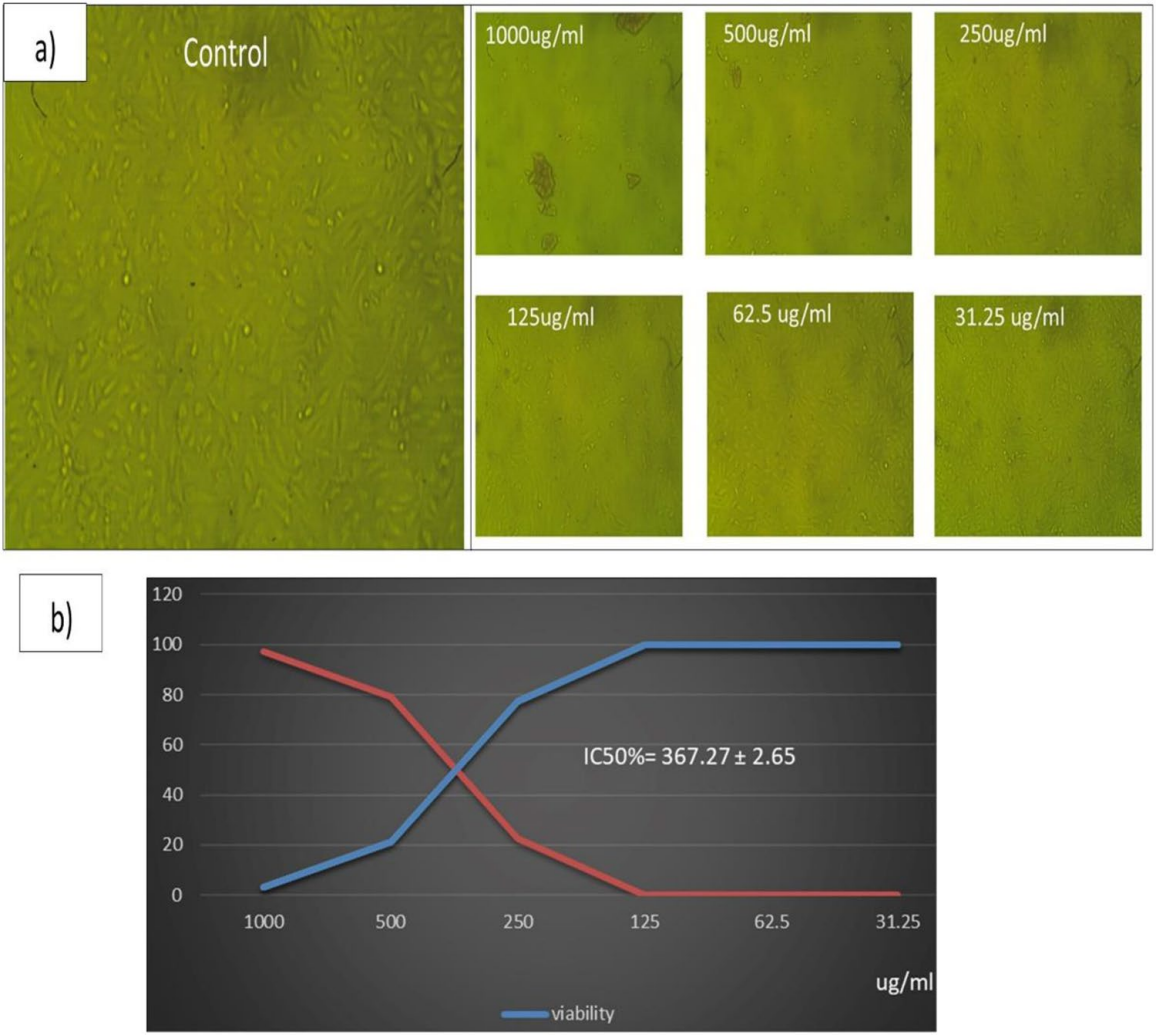
(**a**) Microscopic images of the morphological changes in Vero-CCL-81 cell lines, and (**b**) The IC_50_ of P-CuNPs on ccl-81 cell lines.

**Fig. 9:**
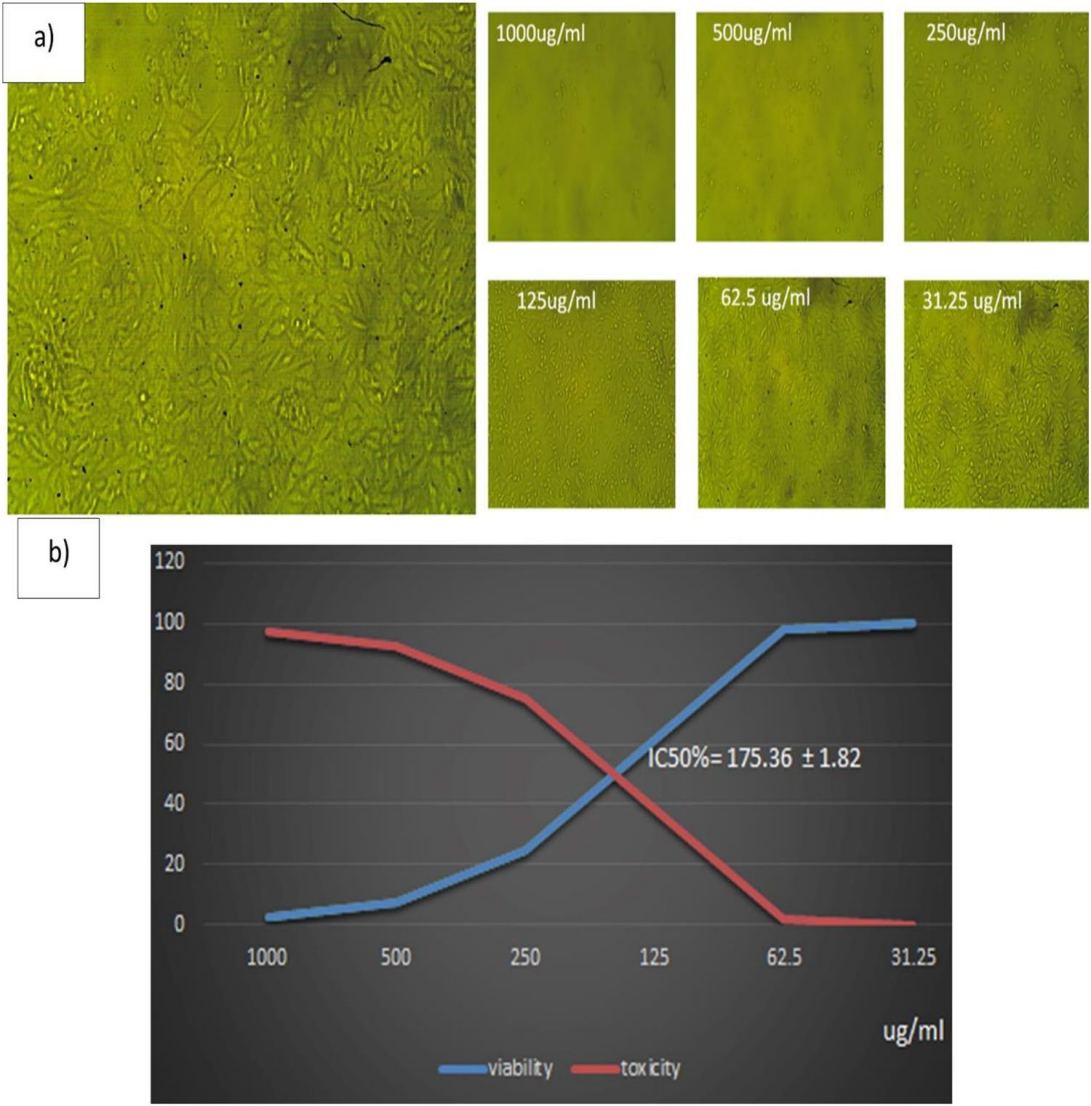
(**a**) Microscopic images of the morphological changes by P-CuNPs as antitumor agent in ATB-37 cell lines, and (**b**) The IC_50_ of P-CuNPs as antitumor agent on Caco-2-HTB-37 cell lines.

Table S3 displays the results of the cytotoxicity determination. The viability of the Vero-CCL-81 cell lines was found to be 2.9, 21, 77, 99.8, 99.9, and 100%, respectively, when exposed to varying doses of P-CuNPs, which were 1000, 500, 250, 125, 62.5, and 31.25 μg/mL. The morphological alterations in ccl-81 cell lines treated to 1000, 500, and 250 μg/ml of P-CuNPs were depicted with microscopic pictures in Fig. 8a. The half-maximal inhibitory concentration (IC_50_) values, which assess the concentration of a pharmacological dosage necessary for 50% of cell death, were computed with the use of the curve. The data shown in Fig. 8b indicates that the P-CuNPs generated on CCL-81 cell lines had an IC_50_ value of 367.27 μg/mL. Using different doses of 1000, 500, 250, 125, 62.5, and 31.25 μg/mL of CuNPs, antitumor study of P-CuNPs against Caco-2-HTB-37 cell lines was created. The viability of ATB-37 cell lines was 2.48, 7.7, 24.8, 61.6, 98, and 99.95%, respectively, as stated in (Table S4) microscopic pictures P-CuNPs were thought to exhibit anticancer action based on the morphological alterations seen in ATB-37 cell lines subjected to 1000, 500, 250, and 125 μg/mL of P-CuNPs, as presented in Fig. 9a. As shown in Fig. 9b, the IC_50_ value of CuNPs was 175.36 μg/mL.

## Discussion

When compared to physical and chemical processes, green synthesis of CuNPs using *P. megaterium* AMD 2024 (PP528505.1) is considered a unique biosynthesis approach because of its environmentally benign nature. The green synthesis procedure of P-CuNPs was confirmed by the color change in the mixture's response to CuSO_4_ combined with *P. megaterium* cell-free extract. Comparably,^8^ noted that the color changed to a greenish hue due to the production of CuNPs by *Bacillus niacini* strain CP-2MN. The similar hue shift was observed by^29^ with *B. megaterium*. Similar studies of^20,30^ employed *Pseudomonas putida* and *P. silesiensis* to change the hue to green.

The P-CuNRs' greatest SPR peak was seen at λ max 450 nm, which corresponds with the results obtained by^31^. Conversely,^32^ found that CuNPs biosynthesized by *Enterococcus thailandicus* had a maximum SPR of 569 nm. Using a *Nelumbo nucifera* (lotus) leaf,^33^ demonstrated that the greatest SPR occurred between 250 and 450 nm.

According to^34^, the conversion and stability of the biosynthesized CuNPs may be caused by a number of substances present in the cell-free supernatant, which might account for the results of these experiments. Additionally, during *B. magisteriu*m’s green synthesis of AgNPs,^29^ examined the interaction between the medium elements and cell-free extract. The strength of the acquired SPR peak was scored at the ideal wavelength, which was a strong indicator for the bioconversion of copper ions to nanoparticles in the reaction mixture, thus providing assurance of the biosynthesis process^20^.

The circumstances of biosynthesizing (temperature, shaking speed, incubation duration, and metabolites for reduction of CuSO_4_) affect the particle size of the biosynthesized CuNPs, varying based on the variation among the green synthesized techniques. *P. megaterium* was used in this investigation, and after 48 h at 30 °C and 150 rpm shaking flask, the particle size of P-CuNPs was 57.73 nm. On the other hand,^35^ reported that the particle size, utilizing *Bacillus* DPS RE RSCIC 24, was 53.2 nm after 48 h at 22 °C and 150 rpm in shaking flasks.

It was discovered that 48 h of incubation was the ideal duration for obtaining the desired concentration by examining the time course of the green produced P-CuNPs. Following this time, the concentration of the biosynthesized CuNPs did not rise. In agreement with^36^, who discovered that 48 h is the ideal window of time to obtain a high concentration of CuNPs while working with *Bacillus* sp. On the other hand,^37^ reported that the ideal duration for the synthesis of CuNPs using *Bacillus cereus* was found to be 79 h. This discrepancy in time might potentially be attributed to other parameters regulating the biosynthesis conditions, which are not examined in this work. The time course results corroborate the findings of^38^, who reported that nanoparticle creation occurs during a certain time period, following which the concentration and particle size remain constant as a result of non-agglomeration.

The best byproduct and carbon source was blackstrap sugarcane molasses, whose size was reduced by 94.6% to 3.122 nm. In contrast, the waste from banana peels had the biggest particle size, measuring 105.7 nm, with a size increase of 83.09%^20^ found that blackstrap sugarcane molasses, with a size of 80.5 nm, was the most appropriate carbon source for the formation of CuNPs using *P. silesiensis* strain A3, as opposed to glucose, which had a size of 87.1 nm. However, because of its size of 3.122 nm, which was lowered by 94.6% utilizing *B. megaterium* FNCC 0083, the material utilized in this investigation was the most appropriate carbon source. Using the aqueous extract of *Commelina nudiflora*, the particle size of the green produced CuNPs varied from 45 to 100 nm^39^.

Additionally, as documented by^40^, the best sources of biosynthesis for AgNPs generation were molasses and brewery liquor inoculated with *B. subtilis* T-1cell-free supernatant. In the similar vein^41^, demonstrated how *Chaetomium globosum* may use a variety of agro-industrial wastes, including olive cake waste and peels from apples, carrots, and potatoes, in the production of zinc nanoparticles. The superior quality of blackstrap sugarcane molasses may be ascribed to its abundance in sugars, vitamins, and other growth factors that are necessary for the development of proteins, secondary metabolites, enzymes, and microorganisms^40^. Additionally, it was reported that the thickness of the production medium, which has a negative impact on culture aeration—a critical component for bacterial growth, the production of secondary metabolites, and reduction enzymes—may be the cause of the reduction in CuNPs concentration when using various agro-industrial wastes, such as broken rice, olive cake, sugarcane bagasse, and sugar beet waste^42^.

P-CuNPs' form and morphology were examined using HR-TEM. *P. megaterium* cell free extract components coat the sphere-shaped biosynthesized P-CuNPs in this work.^37^ demonstrated that *Bacillus cereus* produced amorphous, spherical copper nanoparticles by biosynthesis. *Bacillus coagulans* (GBI-30, 6068) was used to create spherical copper oxide nanoparticles (NPs)^43^.

According to the XRD analysis, the samples were well-crystallized and devoid of any copper oxides or CuSO_4_ impurities. According to^44^, the biosynthesis of CuONPs using *Brevibacillus brevis* MT994671 resulted in diffraction peaks with 2θ at 32.62°, 35.6°, 38.68°, 48.8°, 53.68°, 58.4°, 61.58°, and 66.48°, respectively. These correspond to plane peaks of (110), (002), (111), (020), (202), (113), and (310), and it was also noted that the biosynthesized CuONPs had high purity.

The functional groups in the P-CuNPs cell-free extract that ranged from 500 to 4000 cm^-1^ were investigated using FTIR analysis. Peak locations were found to be between 412.692 and 3860.79 cm^-1^. Alkene, alcohol, carboxylic acid, carbon dioxide, isothiocyanate, amine, alkane, benzene derivative, and halo compound were shown to be the active functional groups, in that order. C–H, O–H, O = C = O stretching, N = C = S stretching, N–H bending, C–H bending, C–N stretching, C–CL, C–Br, and C–L stretching were shown to be the active functional groups. According to the results of FTIR analysis, proteins in particular were in charge of reduction and capping, whereas other substances, such as fatty acids, were in charge of stabilizing using *Ulvophyte* sp. MBIC10591 and *Coelastrella aeroterrestrica* (Strain BA_Chlo4), respectively^45,46^. The functional groups found in the *Bacillus coagulans* filtrate (GBI-30, 6068) (N = C = S, O–H, N–H, and C–H) are the same as those found in the current study, with some variations with respect to other functional groups^43^.

It is possible to conclude from the EDX and XRD analyses that P-CuNPs and *P. megaterium* growth on blackstrap sugarcane molasses have a substantial molecular link. The FTIR study reveals bending amide and amine stretching functional groups, indicating the presence of proteins such protein coating and Cu reductase enzyme that are in charge of the reduction and stability of CuNPs^20^. The reduction and anti-oxidation action that increases the P-CuNPs stability is caused by these proteins that coat the P-CuNPs^20,47^.

According to^46^, the zeta potential examination of P-CuNPs revealed a value of − 11.0 mV, suggesting that the biosynthesized CuNPs utilizing *P. megaterium* were generally stable and had a nonionic character in the cell-free supernatant of the capping molecules. According to another work, the biosynthesized CuNPs utilizing *Bacillus* DPS RE RSCIC 24 had a zeta potential charge of -25.1 mV^35^. According to^38^ the CuNPs' unique and desirable zeta potential negative charge is most likely a result of their protein covering. A long shelf life and great stability were also provided by the negative charge zeta potential, which was discovered to generate repulsion among the CuNPs and inhibit agglomeration and aggregation due to electrostatic stabilization^20^.

The previous studies concurred with^21,48^ demonstrated that Gram negative bacteria were more resistant to CuNPs than gram positive bacteria which more succbiptable. This may be due to differences in the structure of bacteria's cell walls. Furthermore, periplasmic protein, or CueP, is a component of the Cue system that is abundant in many Gram negative bacteria and is essential for their survival in copper-rich settings as well as their ability to resist copper^49^. CuNPs attack bacteria by generating a lot of reactive oxygen species (ROS), which oxidizes fats, breaks down DNA, and ultimately causes the cells to perish^50^. Additionally, CuNPs interact with intracellular components (DNA bases, proteins) and bind to phosphorous and sulfur-containing macromolecules in cellular membrane proteins, hence influencing all metabolic processes (cell division, respiration, and cell survival). On the other hand, some studies have shown that Cu^+^ attaches to amino groups and thiols in proteins, inhibiting and disrupting protein structures. It is commonly recognized that the generation of reactive oxygen species (ROS) by thiol groups results in the inhibition of respiratory enzymes and, ultimately, in mortality. So copper ions are important antibacterial agents because they interact with peptidoglycan in the plasma membrane and cell wall and stop the reproduction of bacterial DNA by interacting with sulfhydryl groups in proteins^20^.

P-CuNPs' cytotoxicity on Vero-CCL-81 cell lines was measured at IC_50_ 367.27 µg/mL, indicating that they might be employed in a range of applications from this dose to ones that would be safe for the environment and humans. P-CuNPs' antitumor evaluation on the viability of ATB-37 cell lines revealed that CuNPs worked in a dose-independent way, as evidenced by a rise in the antiproliferative profile with increasing CuNPs concentration^21^. P-CuNPs shown good antiproliferative activity with an IC_50_ of 80.78 µg/mL when^51^ studied their anticancer activity for human acute T-cell leukemia cell lines.

## Conclusion

Finally, the work sought to develop an environmentally friendly method of biosynthesizing CuNPs from *P. megaterium* AMD 2024 cell-free extract by using agro-industrial wastes and by-products. P-CuNPs were biosynthesized within 450 nm at an SPR of 0.58 and a particle size of 57.73 nm using a fermentation basal glucose medium, as validated by UV–visible spectroscopy. The most productive agro-industrial waste was found to be blackstrap sugar cane molasses, which had the lowest particle size (3.122 nm) in an experiment to replace the expensive medium with other agro-wastes and by-products. P-CuNPs were stable and possessed a sphere-like form. Gram positive bacteria were the most pathogenic bacteria that were susceptible to P-CuNPs. When applied to colon cancer cells, P-CuNPs had a strong antitumor impact; for normal kidney cells, the IC_50_ value was 367.27 μg/mL. In the future, P-CuNPs will be utilized as an antibacterial agent to reduce the microbial burden and resistant in many sources, including contaminated food, water, and surfaces.

## Ethical approval

There are no studies of this article that include human subjects or animals.

## Supplementary Information

Supplementary Information.

## Data Availability

The authors state that every piece of information gathered and examined for this study is included in the publication. The following strain suppliers received all of the microbial pathogens from the Agricultural Microbiology Department of the Faculty of Agriculture at Ain Shams University in Cairo, Egypt. *B. cereus* ATCC 11778 was from the ATCC collection https://www.atcc.org/products/11778. *S. aureus* ATCC 6538 was from the ATCC collection https://www.atcc.org/products/6538. *E. coli* ATCC 8739 was from https://www.atcc.org/products/8739. *P. aeruginosa* ATTC 27853 was from ATTC collection https://www.atcc.org/products/27853. *Priestia megaterium* AMD 2024 strain was identified and deposited in gene bank https://www.ncbi.nlm.nih.gov/nuccore/PP528505.
