# Supplementary material for: Copper nanoparticles biosynthesis by Priestia megaterium and its application as antibacterial and antitumor agents
Source: Sci Rep. 2024 Oct 9;14:23615. doi: 10.1038/s41598-024-72598-3 (PMC11464900; doi:10.1038/s41598-024-72598-3)
Supplement: Supplementary file 1 — Supplementary Information. [file 41598_2024_72598_MOESM1_ESM.pdf]

Supplementary data

Journal: Scientific reports

Copper nanoparticles biosynthesis by *Priestia megaterium* and its application as antibacterial and antitumor agents

Salma H. Mohamed[1], Badawi A. Othman[1], Basma T. Abd-Elhalim[1*], Mohammed N. Abou Seada[1]
[1]Department of Agricultural Microbiology, Faculty of Agriculture, Ain Shams University, Hadayek Shoubra, PO Box 68, Cairo 11241, Egypt.
***Corresponding author: Basma T. Abd-Elhalim (B.A)**
basma.talaat@agr.asu.edu.eg, dr.basma.talaat2020@gmail.com
ORCID: 0000-0002-3009-332X
Phone: +201007025808

Author information

1- Salma H. Mohamed
Affiliation: Department of Agricultural Microbiology, Faculty of Agriculture, Ain Shams University, Hadayek Shoubra, PO Box 68, Cairo 11241, Egypt.
E-mail: salmahesham@agr.asu.edu.eg
ORCID: 0009-0003-3086-4096
Phone number:+201010138737

2- Badawi A. Othman
Affiliation: Department of Agricultural Microbiology, Faculty of Agriculture, Ain Shams University, Hadayek Shoubra, PO Box 68, Cairo 11241, Egypt.
E-mail: badawi_osman@agr.asu.edu.eg
Phone number:+201284664355

3- Basma T. Abd-Elhalim* (Corresponding author)
Affiliation: Department of Agricultural Microbiology, Faculty of Agriculture, Ain Shams University, Shubra El-Khaimah, Cairo 11241, Egypt.
E-mail: basma.talaat@agr.asu.edu.eg
ORCID: 0000-0002-3009-332X
Phone number:+201007025808

4- Mohammed N. Abou Seada1
Affiliation: Department of Agricultural Microbiology, Faculty of Agriculture, Ain Shams University, Hadayek Shoubra, PO Box 68, Cairo 11241, Egypt.
E-mail: abouseada@agr.asu.edu.eg
Phone number:+201223139172

Table 1S. Collective sources of agro-industrial wastes and by-products.

Agro-industrial wastes and byproducts	Source
1- Arish cheese whey	Local market, Shoubra-El Khima, Cairo.
2- Banana peel	Local market, Shoubra-El Khima, Cairo.
3- Blackstrap sugar beet molasses	Delta sugar company, Kafr El sheikh.
4- Blackstrap sugarcane molasses	Sugar refinery factory, El-Hawamdia.
5- Sugar beet waste	Delta sugar company, Kafr El sheikh.
6- Sugar cane bagasse	Local sugar cane juice shop, Shoubra-El Khima, Cairo.

Table 2S. Antibacterial activity as inhibition zone diameter of Pm-CuNPs on pathogens bacteria.

Pathogen bacterial strains	Inhibition zone (mm)							
	kanamycin (1000 µg/ml)	Concentrations of P-CuNPs (µg/ml)						
		1000	500	250	125	62.5	31.25	AI
***B. cereus* ATCC 11788**	13.0[c] ±0.08	26.0[b] ±0.33	24.0[c] ±0.18	18.0[f] ±0.33	14.0[g] ±0.87	9.0[i] ±0.2	4.0[l] ±0.18	2.0
***S. aureus* S ATCC 6538**	18.2[a] ±0.07	28.0[a] ±0.2	23.0[cd] ±0.39	21.0[e] ±0.75	14.0[g] ±0.16	11.0[hi] ±0.4	8.0[ij] ±0.27	1.54
***E. coli* ATCC 8739**	14.0[b] ±0.2	12.0[h] ±0.21	8.0[i] ±0.4	6.0[k] ±0.24	6.0[k] ±0.11	0.0	0.0	0.86
***P. aeruginosa* ATCC 27853**	12.0[cd] ±0.05	0.0	0.0	0.0	0.0	0.0	0.0	0.0

AI = Activity index. Values are means ± SD (n = 3). Data within all groups are analyzed using ANOVA by Duncan's test [28].

Table 3S. Cytotoxicity activity of Pm-CuNPs with various concentrations against ccl-81cell lines.

ID	ug/ml	O.D			Mean O.D	±SE	Viability %	Toxicity %	IC50 ± SD
**ccl-81cell lines**	--------	0.717	0.719	0.712	0.716	0.002	100	0	ug
**Pm-CuNPs**	1000	0.018	0.025	0.02	0.021	0.002	2.90	97.06	367.27 ± 2.65
	500	0.163	0.138	0.152	0.151	0.007	21.0	78.9	
	250	0.54	0.562	0.558	0.55	0.006	77.0	22.7	
	125	0.711	0.719	0.715	0.715	0.002	99.8	0.10	
	62.5	0.716	0.72	0.71	0.715	0.002	99.9	0.09	
	31.25	0.718	0.714	0.716	0.716	0.001	100	0.00	

Table 4S. Antitumor activity of Pm-CuNPs with various concentrations against ATB-37cell lines.

ID	ug	O.D			Mean O.D	±SE	Viability %	Toxicity %	IC_{50} ± SD
Caco2	--------	0.742	0.739	0.733	0.738	0.0026	100	0	ug
Pm-CuNPs	1000	0.018	0.02	0.017	0.018	0.00088	2.48	97.5	175.36 ± 1.82
	500	0.064	0.048	0.059	0.057	0.0047	7.70	92.0	
	250	0.184	0.169	0.197	0.18	0.008	24.8	75.0	
	125	0.462	0.458	0.444	0.45	0.005	61.6	38.0	
	62.5	0.726	0.719	0.73	0.725	0.003	98.0	1.76	
	31.25	0.74	0.735	0.738	0.73	0.001	99.95	0.045	
